# Operation makalu air crash: influence of cognitive and human factors on decision-making

**DOI:** 10.1080/20961790.2022.2095691

**Published:** 2023-02-12

**Authors:** Samarika Dahal, Gopal Kumar Chaudhary, Nitin Kumar Agrawal

**Affiliations:** a Department of Dentistry, Institute of Medicine, Kathmandu, Nepal; b Department of Forensic Medicine, Maharajgunj Medical Campus, Maharajgunj, Kathmandu, Nepal

**Keywords:** Forensic sciences, disaster, cognitive bias, dental identification, mortuary, Nepal

## Abstract

Two onboard crew members lost their lives in the fatal Makalu Air Cessna Grand Caravan 208B domestic cargo flight crash on May 16, 2018. The Disaster Victim Identification (DVI) procedure comprises external examination, photography, DNA collection, fingerprint collection, postmortem examination, antemortem information collection from the family members, and reconciliation. The major challenge of this operation was dealing with cognitive bias. The antemortem dental information of one of the deceased was revealed to the forensic experts just before the postmortem examination. This influenced the testing strategies. There was a tendency to neglect the complete dental examination presuming the identification was established. Later, during a thorough examination, the forensic odontologist realised that the initial decision was erroneous. Furthermore, there are few experience-based resources available to resolve cognitive bias issues. The authors begin by summarising complicated operations in which they have been involved, followed by a discussion of the key sources of cognitive bias along with the solution to resolve these issues in DVI preparedness planning.

## Key points

Discussion of Disaster Victim Identification experience by the involved team members

Forensic odontologists discuss about the situation of bias during the operation

This article highlights the importance of adhering to the best practices of disaster victim identification process irrespective of the size of the disaster

## Introduction

The frequent air crashes in Nepal have been a prime concern for the Civil Aviation Authority of Nepal (CAAN). According to Yadav [1], 37 fatal air crashes occurred between 1961 and 2016. The statistics indicate that there is at least one air crash incident each year [2]. Human factors are attributed as the prime cause of these fatal accidents followed by environmental factors, with system factors being the least common cause [2]. Following a disaster, standard practice is to recover all bodies from the scene, pack them in body bags, and transport them to a morgue for the Disaster Victim Identification (DVI) process [3].

DVI is a complex process that relies on two accepted means of identification: primary and secondary. The primary identifiers comprise fingerprint, DNA, and dental comparisons. The secondary identifiers incorporate details of the missing person, personal description, anthropological and circumstantial evidence, and medical procedures done in the past. Fingerprint analysis and dental comparisons are the methods of choice due to their low cost and precision in identification [4,5].

The International Criminal Police Organization (INTERPOL) is an intergovernmental organisation that assists the 195 member countries’ police services in making the globe a safer place. The INTERPOL protocol divides DVI into five phases from the time of recovery until the deceased’s burial/cremation—Phase 1: the disaster scene; Phase 2: the mortuary/postmortem and data collection; Phase 3: antemortem data collection; Phase 4: reconciliation; and Phase 5: debriefing [4]. The guidelines, although not compulsory, are recognised and accepted globally [6]. Nepal has adopted the standard international protocol of INTERPOL [7].

The scientific identification of human remains is based on the principle of matching antemortem data compiled by the antemortem data collection team with the postmortem information collected by the forensic experts in the mortuary [8]. This process may appear quite straightforward but is tricky in reality [9].

Forensic experts may face a variety of challenges associated with data analysis and interpretation in forensic cases. Postmortem examination information may be rendered useless due to the partially or incorrectly retrieved remains from the scene. The completely recovered remains might need re-examination due to missed information during the initial examination. Low visibility, improper lighting, limited mouth opening, and absence of X-ray facilities in the morgue may contribute to discrepancies in charting. Similarly, complexities such as the lack of standard practice for archiving dental records in many countries, incomplete charting of the dental records by dentists, and frequent changes of the treating dentists by the patients makes antemortem dental data retrieval extremely challenging. Inaccurate and incomplete dental records cause distorted transcription information. Even if the antemortem team is successful in retrieving such dental evidence, it may be meaningless during the reconciliation process. The compounded faults encountered in each step of DVI make human identification extremely difficult [9,10].

This article analyses a situation of cognitive bias faced by forensic experts during the DVI operation. The antemortem dental information of one of the deceased was transcribed to the forensic odontologist (FO) in charge of the postmortem examination in the mortuary. Based on a preliminary examination performed out of curiosity, the FO presumed that MAC-001 had been identified based on this key information without conducting a complete examination of both bodies. This would have been a case of misidentification if the forensic experts had deviated from the standard quality assurance protocol. Based on case-based experience, it is recommended that forensic experts working on a single case or, during DVI operations, should adopt best practices for sequential unmasking of the information to avoid biases.

## Case report

On May 16, 2018, a Makalu Air Cessna Grand Caravan 208B on a domestic cargo flight that took off from Surkhet at 06:15 h local time to Simikot went missing for almost 4 h. The plane was reported to have crashed at approximately 10:40 h on the mountainside of Simikot pass at an altitude of 3 900 m. The bodies of both crew members were transferred from the fatal crash site to the Department of Forensic Medicine (DOFM), Maharajgunj Medical Campus, Kathmandu, at 15:15 h on the same day. The department was thus given the authority and responsibility of conducting the DVI. On receiving the bodies, the DVI commander (department head of the DOFM) initiated the DVI operation according to the INTERPOL guidelines. Antemortem, postmortem, and reconciliation teams were formed under the supervision of the respective commanders. This operation involved a total of 11 forensic medicine experts. They were assigned duties at various stations: the external examination (4), postmortem examination (4), and antemortem data collection (3). Two officials from the Central Police Forensic Science Laboratory, Kathmandu, Nepal, collected DNA and fingerprint samples. Two FOs examined the teeth and recorded the information using the Fédération Dentaire Internationale (FDI) tooth numbering system.

The autopsy examination of both the bodies was conducted at the DOFM from 17:00 h to 20:00 h on May 16, 2018. Each body was assigned a unique reference code (MAC-001 and MAC-002). At first, an external examination was conducted, followed by the collection of a DNA sample and fingerprints. After that, a complete autopsy was performed. The cabin crew’s biological samples were also collected to examine if intoxication and/or a medical condition contributed to the fatal plane crash. Lastly, the dental examination was done.

Postmortem examination revealed both the deceased were males, in different age groups. The length of MAC-001 was 1.524 m while MAC-002 was 1.702 m. Before the dental examination, one of the antemortem team members informally disclosed the antemortem dental information to the FO. The presumptive identification of MAC-001 had already been established based on his external appearance, so a dental examination seemed unnecessary. Out of curiosity, the FO quickly examined the oral cavity of MAC-001 and confirmed that the information about the lost upper right posterior teeth conveyed to the FO was correct. In the absence of standard operating procedures, the natural tendency would be to return MAC-001 to the family member without even completing the examination of all the bodies.

However, the standard protocol in DOFM mandates that all the deceased during DVI operations be examined by an FO. Thus, a complete examination of both MAC-001 and MAC-002 was done.

The postmortem dental examination of MAC-001 showed teeth 15, 37, and 47 were missing antemortem. There were age-related changes in the teeth and gingiva, such as tooth abrasion, attrition, and gingival recession, indicating that the deceased was in his later years. Postmortem dental examination of MAC-002 showed tooth 26 was missing antemortem. The oral cavity appeared to be in good health, with no evidence of regressive tooth alteration or age-related changes in the gingiva indicating adulthood. The basis of identification of both MAC-001 from MAC-002 is indicated in Table 1.

**Table 1.** Comparison tools used for the identification of MAC-001 and MAC-002.

Item	MAC-001	MAC-002
Personal belongings		✓
Physical features	✓	✓
Exclusion	✓	

Family interviews were conducted on May 16, 2018. All the details were collected from the immediate family members. During the family interview, one of the families shared information about one missing tooth, but neither of the families could provide dental records. The antemortem records were named FM75-0147 and FM75-0148. The antemortem data of the FM75-0147 indicated that one tooth was missing in the right upper posterior region. The type and location of extracted teeth were unknown to the family members. The antemortem data of FM75-0148 had no dental information. The family was unable to identify the treating dentist. Both families were able to provide citizenship papers, indicating that FM75-0147 was 54 years old and FM75-0148 was 30 years old.

The reconciliation was initiated immediately after the postmortem examinations and completed at 23:00 h. The points of agreement used to determine the identity of the deceased in this closed disaster were physical features and personal belongings. Provision of an age estimate based on the existing teeth conditions corroborated the dental findings. After the board unanimously accepted the findings, the DVI Commander verified the established identification. The FO could have made an error if they had relied on the transcribed antemortem information and neglected the complete dental examination.

## Discussion

The dental findings in this case were initially presented as a basis of identification. There were two bodies, and one family had dental information about the extraction of an upper right posterior tooth. Based on this antemortem dental information, it was speculated that MAC-001 was probably the missing crew member whose dental information was provided. However, the dental examination of MAC-002 revealed a missing upper left first molar. In the absence of the dental records from the treating dentists, the forensic experts could not rely on antemortem information based on family interviews. Thus, this information had to be discarded. It is reasonable for any distressed family to be confused about which side of the jaw was treated. In such circumstances not only the inexperienced dentists/FOs, but even skilled dentists can fall prey to bias leading to misidentification [11].

The term “cognitive bias” refers to a variety of processes that might lead to incorrect judgments or interpretations. Memory, logic, and decision-making can all be influenced by cognitive biases as a result of human judgment distortion [11–13]. Dror [14] has broadly categorised the source of cognitive bias into three groups.

Category A is related to a specific case that causes bias in the way data are viewed, assessed, and comprehended. Category B is not related to a specific case, but the factors related to the expert’s experience, personality, their working environment, and their motivation causing the bias. Category C, related to human nature, is the very cognitive architecture of the human brain.

The FO in the present case experienced all these sources of bias. Before the dental examination, the antemortem data were conveyed to the FO and a presumptive identification was established in the mortuary. The FO initially examined only the right upper posterior region and confirmed that the information was accurate. Best practice for communication between antemortem and postmortem team members was non-existent at that time, contrary to current practice. As a result, their brains received and analysed information differently, exposing them to cognitive bias.

The sources of bias are further subdivided into data, reference material, contextual information, base rate, organisational factors, education and training, personal factors, human and cognitive factors, and the human brain. These eight sources of bias could influence forensic experts in various ways during decision-making [14].

Antemortem data that can contain potentially biasing information can have an impact on decision-making [15]. Misinterpretation of missing teeth is the most common. For example, antemortem data may indicate that the lower right second molar has been extracted. Due to confirmation bias, a mesially migrated lower right third molar may be interpreted as the second molar [16].

Reference materials have an impact on how data is received and interpreted. In this bias, experts start working backwards, letting the target or expected findings drive the process [14,17]. For example, root canal treatment in the lower right first premolar is documented in antemortem dental records. The same was observed during a postmortem dental examination.

Contextual information can bias testing strategies [18]. This type of biased source typically points to straight confirmation and quantification evidence rather than potentially opposing the evidence [14,18]. For example, agenesis of the third molar in both the upper and lower quadrants may be interpreted as extracted third molars based on antemortem dental data of the presumed dead person. The testing procedures may be altered due to the impaired choice abilities caused by confirmation bias. A confirmatory X-ray test may be missed.

Experience from previous cases impacts the expert’s decision and interpretation [14,19]. Uncommon findings during the dental examination are likely to be missed by experts based on past experiences. For example, a notch in the maxillary central incisor is a rare finding. If present in the presumed dead individual, it is likely to be missed by an FO during postmortem dental examination.

The organisational component also plays a big role [14]. Junior teammates are less likely to challenge the decision if a senior FO confirms the results. As a result, science is now tied closely to organisational power and a diverse variety of other challenges [20].

Education and training are critical in terms of how work is done. A dentist trained in forensics compared with an FO may approach and make conclusions about a case differently. Also, over time, an individual’s experience and formal education enable them to make categorical decisions about whether to accept or reject pieces of evidence [14].

Other factors include human and personal factors such as the risk-taking or avoidance nature of the individual. The ability to withstand extreme work pressure and fatigue during DVI operations may influence decision-making capacity [14,21].

To minimise bias in human identification it is vital to practise sequential unmasking [22] and linear sequential unmasking [23]. Optimising the order of information not only reduces distortion and improves overall decision quality, but also reduces bias. For determining the appropriate sequence of task-relevant information exposure, Dror and Kukucka [22] suggest three criteria: biasing power, objectivity, and relevance, which are discussed further below.

Biasing power: Relevant information has a wide range of biasing power. It is recommended that non-biasing (or less biasing) relevant information be exposed first, followed by more strongly biasing relevant information [22]. An example of strongly biasing information is asking the family members leading questions during the antemortem interview, such as “did the deceased have his upper right tooth extracted?” Similarly, during a postmortem examination, revealing of key antemortem information like spacing in upper anterior tooth can pose bias.

Objectivity: Task-relevant data objectivity also varies [22]. Dental charting, for example, is frequently less objective than a video clip or photograph of the relevant finding during a postmortem examination. Completeness, perspective, quality, and other elements can affect the objectivity of the recordings or images. As a result, more objective information should be exposed first, followed by the information that is less objective.

Relevance: Some relevant information is fundamental and is required to support the decision, while other relevant information is less central or not essential [22]. For example, antemortem dental information about the type and materials used for fillings and crowns is essential and should come first rather than the previous history of scaling and gum disease. The more important information should come first, followed by the less relevant information, and any information that is completely irrelevant to the decision (such as tooth bleaching) should be excluded entirely.

Another important consideration to reduce bias in countries with limited resources is to adopt the integrated reconciliation method. This will evaluate all the relevant information such as the stature, biological sex, and personal belongings in a holistic manner to minimise errors [24].

## Conclusion

The FO may face cognitive bias during human identification; therefore, sequential unmasking and linear sequential unmasking should be adopted and practised to minimise bias. The inclusion of this quality assurance protocol may hugely reduce bias due to checks and balances. Additionally, many jurisdictions do not mandate dental examination or enlist FOs/dentists as team members in DVI operations. In such cases, external examination and DNA and fingerprint analysis may be preferred over dental examination, which might lead to erroneous decisions.

In the present case, the absence of an upper right posterior tooth may appear to be adequate to confirm the presumed identification, yet it is unscientific and defies all human identification laws. Such practices should be abolished, and mandatory complete dental examination practices should be adopted to reduce cognitive bias.
